# Supplementary material for: Biomolecular Evaluation of Lavandula stoechas L. for Nootropic Activity
Source: Plants (Basel). 2021 Jun 21;10(6):1259. doi: 10.3390/plants10061259 (PMC8234844; doi:10.3390/plants10061259)
Supplement: Supplementary file 1 [file plants-10-01259-s001.zip › plants-1224126-supplementary.pdf]

plants

Article

Biomolecular Evaluation of *Lavandula stoechas* L. for Nootropic Activity

Aamir Mushtaq [1,2], Rukhsana Anwar [1], Umar Farooq Gohar [3], Mobasher Ahmad [1,2], Marc Romina Alina [4,*], Muresan Crina Carmen [4], Marius Irimie [5,*] and Elena Bobescu [5]

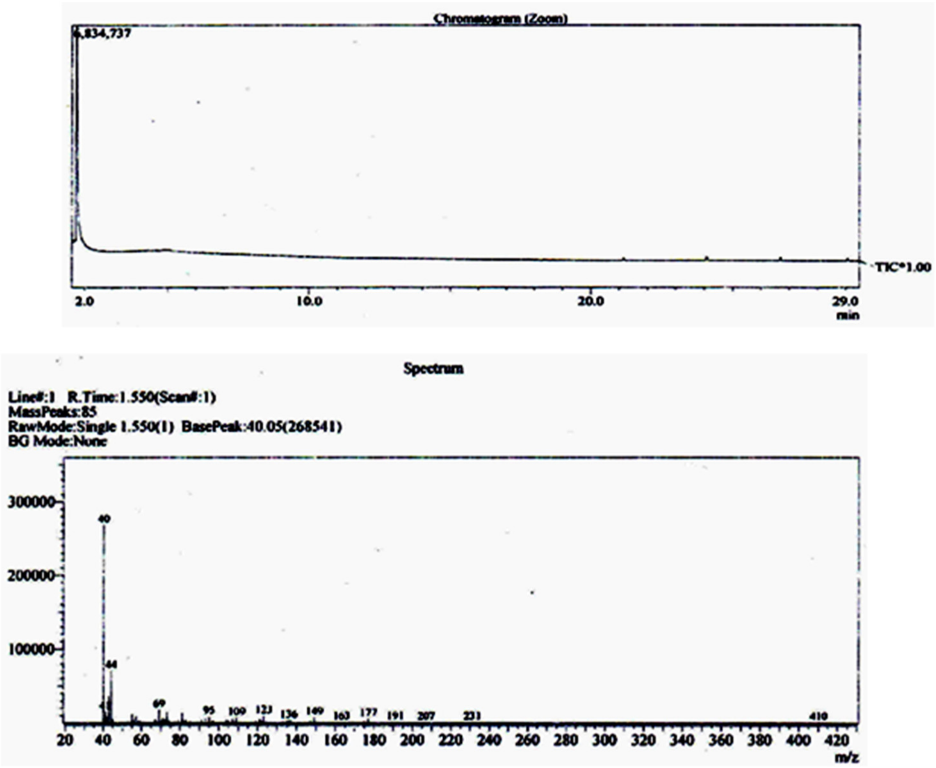

Figure S1. Spectrum of analysis of AfL.s by GC-MS.
